# Research on Fracture Behavior of Fiber–Asphalt Mixtures Using Digital Image Correlation Technology

**DOI:** 10.3390/ma16216825

**Published:** 2023-10-24

**Authors:** Bo Li, Yangyang Zhou, Aihong Kang, Keke Lou, Qianli Gu

**Affiliations:** 1 College of Civil Science and Engineering, Yangzhou University, Yangzhou 225127, China; libo@yzu.edu.cn (B.L.); mx120210598@stu.yzu.edu.cn (Y.Z.); lkkyzu@163.com (K.L.); gqlyzu@163.com (Q.G.); 2 Research Center for Basalt Fiber Composite Construction Materials, Yangzhou 225127, China

**Keywords:** asphalt mixture, fiber type, fiber content, digital image correlation technology, cracking resistance, fracture behavior

## Abstract

Many researchers use fiber to improve the cracking resistance of asphalt mixtures, but research concerning the effects of fiber on fracture behavior is limited. The fracture behavior of asphalt mixtures with various fiber types (basalt fiber, glass fiber, and polyester fiber) and contents (0.1%, 0.2%, 0.3%, 0.4%, and 0.5%) has been studied using the indirect tensile asphalt cracking test (IDEAL-CT) in conjunction with digital image correlation (DIC) technology. The evaluation indexes used in the test included crack initiation energy (*G_if_*), crack energy (*G_f_*), splitting tensile strength (*R_T_*), cracking tolerance index (*CT_index_*), and the real-time tensile strain (*E_xx_*) obtained using digital image correlation technology. The results showed that despite the fiber type, the increase of fiber content resulted in first, an increase, and then, a decrease of the cracking resistance of asphalt mixtures, indicating the presence of optimum fiber content—specifically, 0.4%, 0.3%, and 0.3% for basalt fiber, glass fiber, and polyester fiber, respectively. The development of real-time tensile strain, obtained based on digital image correlation technology, could be divided into two stages: slow-growth stage and rapid-expansion stage. In addition, asphalt mixture with basalt fiber presented the best cracking resistance at both the slow-growth and rapid-expansion stages. This research is helpful in understanding the effects of fiber type and content on the fracture behavior of asphalt mixtures and has certain reference significance for the application of fiber in asphalt mixtures.

## 1. Introduction

Because of long-term effects such as temperature stress and driving load, asphalt pavement is prone to cracking, resulting in surface and even structural damage [1]. Adding fibers into asphalt mixtures can improve their cracking resistance to varying degrees. At present, there are many types of fibers used in asphalt mixtures, and the commonly used fibers include basalt fiber, glass fiber, and polyester fiber. Basalt fiber has the characteristics of high-temperature resistance, high-tensile strength, stable chemical, and environmentally friendly properties. Basalt fiber can improve the high-temperature performance and cracking resistance of asphalt mixtures [2,3], and glass fiber and polyester fiber can also improve the cracking resistance to different degrees [4,5,6,7]. S. Pirmohammad et al. conducted semi-circular bending tests on asphalt mixtures with basalt fiber contents of 0.1%, 0.2%, and 0.3%, and found that the fracture toughness increased with increasing fiber content [8]. S. Qian et al. conducted direct tensile tests on aramid fiber and polyester fiber–asphalt binder and found that adding polyester fiber led to higher tensile ductility of asphalt binder [9]. 

The cracking resistance of asphalt mixtures is affected by the fiber type and content. However, the effects of these two factors on the cracking resistance have not been investigated systematically. Though the effects of fiber types have been analyzed, the limitations in these studies could be that the comparison and analysis of asphalt mixtures with different fibers were commonly based on the same fiber content [10,11], rather than the optimum content with the best improvement effect. In addition, previous studies concerning the effects of fiber content on the cracking resistance of asphalt mixtures often used insufficient types of selected fiber content [8,10], which can be appropriately increased when determining the optimum fiber content.

The main test methods for evaluating the cracking resistance of asphalt mixtures include the indirect tensile asphalt cracking test (IDEAL-CT) [12,13,14], single-edge notch beam (SENB) test [15,16], semi-circular bending (SCB) test [17,18], disk-shaped compact tension (DCT) test [19,20] and so on. Through detailed analysis of the load–displacement curve of indirect tensile asphalt cracking test, many researchers have obtained crack energy, maximum displacement, post-peak slope, and other indexes to evaluate the cracking resistance of asphalt mixtures [21,22]. For the analysis of the cracking resistance of asphalt mixtures, various macro-performance test methods are mainly used—one (or more) index value has been obtained for a certain specimen [12,17,21]—but some of these methods are lacking to be used for the evaluation and analysis of the whole cracking process of asphalt mixtures. The mixtures undergo deformation during the cracking process, accompanied by the fluctuation of the strain. However, the strain distribution of asphalt mixtures cannot be studied by the various macro-performance test methods only, and it is necessary to use image technology. Digital image correlation (DIC) technology was proposed in the 1980s [23,24], which is mainly used to measure surface deformation [25,26,27]. Y. Seo et al. compared the traditional measurement results with those obtained using digital image correlation technology and found that the results obtained by full-field measurement with digital image correlation technology were more reliable and determined the stress–strain behavior accurately [28]. E. Romeo et al. conducted bending tests on asphalt mixture beam specimens and accurately analyzed the distribution of damage and strain using full-field strain diagrams obtained using digital image correlation technology [29]. H. Radhi Radeef et al. conducted a study on the mechanical response of polymeric waste-modified asphalt mixtures, and the resistance curve based on cumulative fracture energy was obtained using digital image correlation technology [26]. Many researchers have incorporated digital correlation technology into macro-performance tests of asphalt mixtures to obtain strain fields during the cracking process for further investigation, and the technology has been proven to be effective and feasible.

This research adopted multiple contents (0.1%, 0.2%, 0.3%, 0.4%, and 0.5%) of basalt fiber, glass fiber, and polyester fiber to fabricate the fiber-modified asphalt mixtures. And the effects of fiber content and fiber type on the cracking resistance of asphalt mixtures were investigated to determine the optimum fiber content and fiber type. In addition, the real-time tensile strain was obtained synchronously in conjunction with digital image correlation technology to further explore the fracture behavior of fiber-modified asphalt mixtures, and the effects of fiber content and fiber type on fracture behavior were clarified. The conclusions of this study can help to understand the fracture behavior of fiber–asphalt mixtures and provide guidance for the application of fibers in asphalt mixtures, such as the selection of fiber content and fiber type.

## 2. Materials and Methods

### 2.1. Materials

#### 2.1.1. Raw Materials

**Figure 1:**
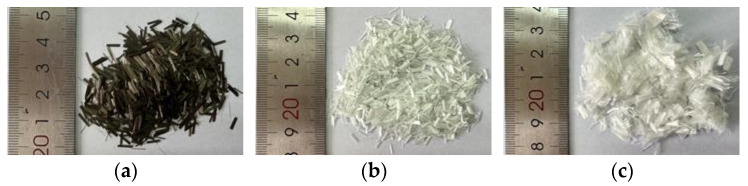
Macroscopic morphologies of fibers: (**a**) basalt fiber; (**b**) glass fiber; and (**c**) polyester fiber.

In this research, SBS-modified asphalt was used. The results of asphalt indexes are shown in Table 1. Limestone was selected for coarse and fine aggregates. Basalt fiber (BF), glass fiber (GF), and polyester fiber (PF) were selected, with the performance indexes shown in Table 2 and the macroscopic morphologies shown in Figure 1. The fibers were mixed into asphalt mixtures at five types of content: 0.1%, 0.2%, 0.3%, 0.4%, and 0.5%, by weight of the total asphalt mixtures.

#### 2.1.2. Gradation Design

**Figure 2:**
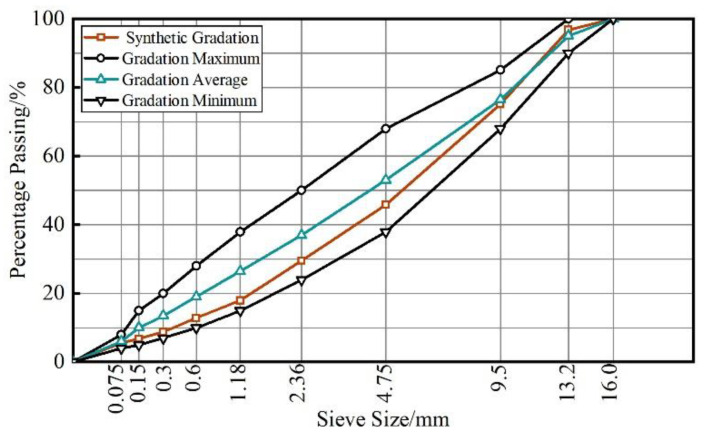
Gradation curve of AC13 asphalt mixture.

Dense-graded asphalt mixture with a nominal maximum aggregate size of 13.2 mm, named AC-13 was selected. The designed gradation curve is shown in Figure 2. The optimum asphalt content of mixtures was determined using the Marshall design method, and the results are summarized in Table 3. It can be seen from Table 3 that, compared with the mixture with no fiber, with increasing fiber content, the optimum asphalt content increased by 0.1 to 0.3 percentage points for BF- or GF-modified mixture, while that of PF-modified mixture increased by another 0.1 percentage point.

### 2.2. Methods

#### 2.2.1. Indirect Tensile Asphalt Cracking Test

**Figure 3:**
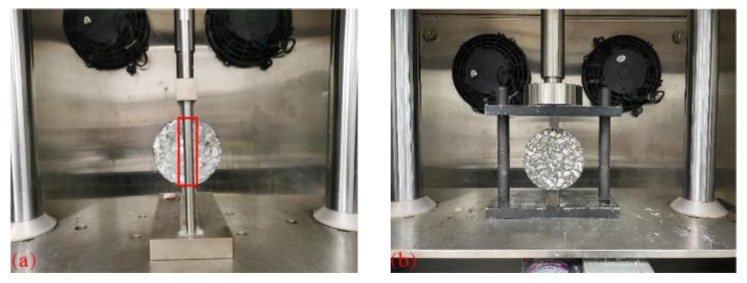
Test fixture: (**a**) original fixture; and (**b**) new fixture.

The indirect tensile asphalt cracking test (IDEAL-CT) developed by the Texas Transportation Institution was used to study the cracking resistance of fiber–asphalt mixtures [30]. In this test, the original cylindrical specimens with a diameter of 150 mm and a height of 150 mm were prepared by the rotary compacting instrument first, and then specimens with a diameter of 100 mm and a thickness of 62 mm were obtained by drilling and cutting. The test was conducted at 25 °C, with the loading head displacement rate of 50 mm/min. Four duplicates were prepared for each type of asphalt mixture specimen. In order to avoid the shadows caused by the left or right cylindrical rods, the original fixture (Figure 3a) was redesigned (Figure 3b) for the photographing process of the fracture surface of the specimen during the test.

**Figure 4:**
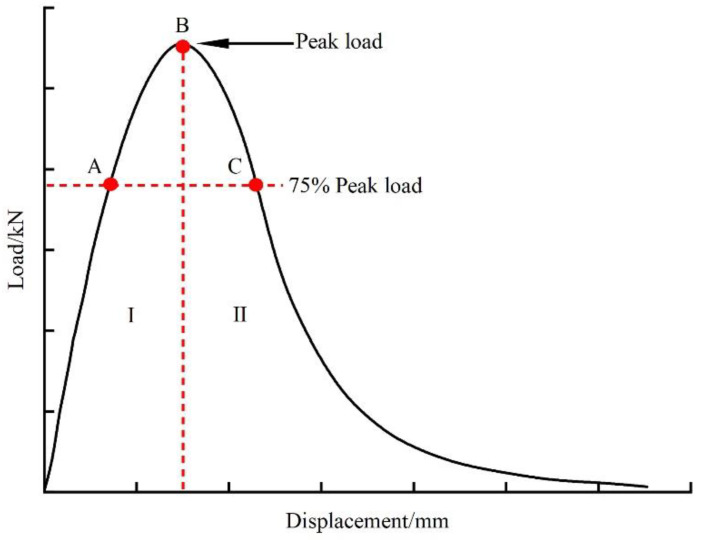
Load–displacement curve diagram.

The load–displacement curve diagram is shown in Figure 4, the whole cracking process can be divided into two stages: resistance to crack initiation (I) and resistance to crack propagation (II). The crack initiation work is the area enclosed by the load–displacement curve and the horizontal coordinate before the peak load, expressed as *W_if_*. The crack initiation energy is the crack initiation work per unit area of the failure section, expressed as *G_if_*. The bigger the values of *W_if_* and *G_if_*, the more work and energy required to form macroscopic cracks in asphalt mixtures. The crack work (*W_f_*) is the area enclosed by load–displacement curve and horizontal coordinate in the whole cracking process, which can represent the work conducted in the whole cracking process of mixtures. The crack energy (*G_f_*) is the crack work per unit area of the failure section, which can represent the resistance of mixtures during the whole cracking process.

The splitting tensile strength (*R_T_*) can reflect the mechanical deformation characteristics of materials, so it can be used to study the deformation characteristics of fiber–asphalt mixtures. The calculation formula is shown in Equation (1).

(1) $$R_{T}=0.006287\times P_{T}/h$$

where *R_T_* is the splitting tensile strength, MPa; *P_T_* is the peak load, N; and *h* is the height of the specimen, mm.

**Figure 5:**
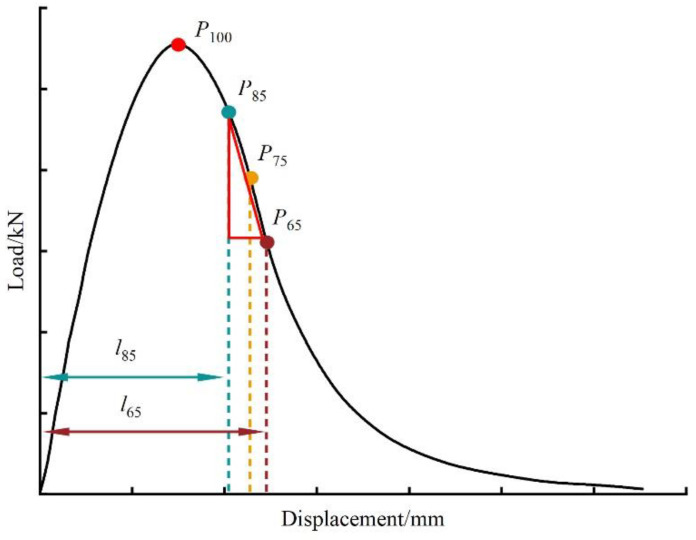
Schematic diagram of |*m*_75_|.

The cracking tolerance index (*CT_index_*) is an evaluation index of the indirect tensile asphalt cracking test, which is derived from the Paris formula. Its variability is small, and it can represent the resistance to crack expansion of asphalt mixtures stably. The larger the *CT_index_* value is, the slower the crack expansion speed and the better the resistance to crack expansion. The calculation formulas are shown in Equations (2) and (3).

(2) $$CT_{index}=\left(t/62\right)\times \left(G_{f}/\left|m_{75}\right|\right)\times \left(l_{75}/D\right)$$

(3) $$\left|m_{75}\right|=\left|\left(P_{85}-P_{65}\right)/\left(l_{85}-l_{65}\right)\right|$$

where *CT_index_* is the cracking tolerance index; *t* is the thickness of the specimen, mm; *G_f_* is the crack energy, J·m^−2^; *l*_65_, *l*_75_, and *l*_85_ is the displacement at 65%, 75%, and 85% peak load, respectively, in the rear section of the load–displacement curve, mm; |*m*_75_| is the slope at the 75% peak load in the rear section of the load–displacement curve, and its schematic diagram is shown in Figure 5; *D* is the diameter of the specimen, mm; and *P*_65_, *P*_85_ is the 65%, 85% peak load, kN.

#### 2.2.2. Digital Image Correlation Technology

Digital image correlation (DIC) technology is used to analyze the gray value before and after the image deformation, to obtain the strain change of the specimen surface. There were natural speckles on the surface of asphalt mixture specimens, and there were differences in gray values between speckles. Digital image correlation technology located the subregions with the same gray value before and after deformation, and calculated the displacement change and strain distribution by correlation method. The steps of using digital image correlation technology in indirect tensile asphalt cracking test are as follows.

Step 1: specimen surface treatment

**Figure 6:**
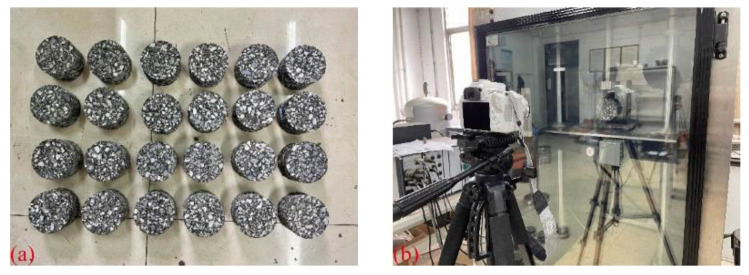

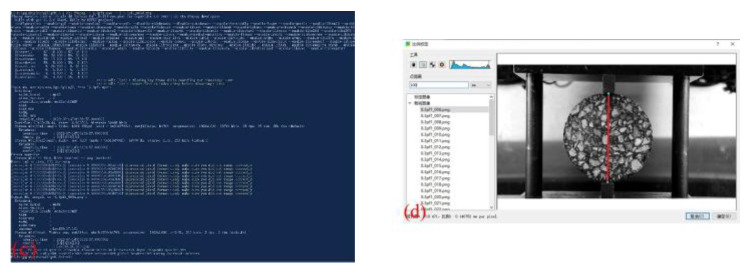
The steps of using DIC technology: (**a**) specimen surface treatment; (**b**) video recording; (**c**) frame taking; and (**d**) image analysis.

The surface of the specimen was slightly polished with a sander and cleaned with a towel, so that the surface of the specimen was smooth and had a clear natural texture, as shown in Figure 6a.

Step 2: video recording and frame taking

The cracking process in the indirect tensile asphalt cracking test was captured synchronously by the camera, as shown in Figure 6b. After recording, ffmpeg software was used to take frames from the video, as shown in Figure 6c.

Step 3: software using

The images taken from the whole cracking video were processed by using DIC technology software. The main steps were image import, AOI region determination, image scale calibration, parameter setting, and image analysis, as shown in Figure 6d.

**Figure 7:**
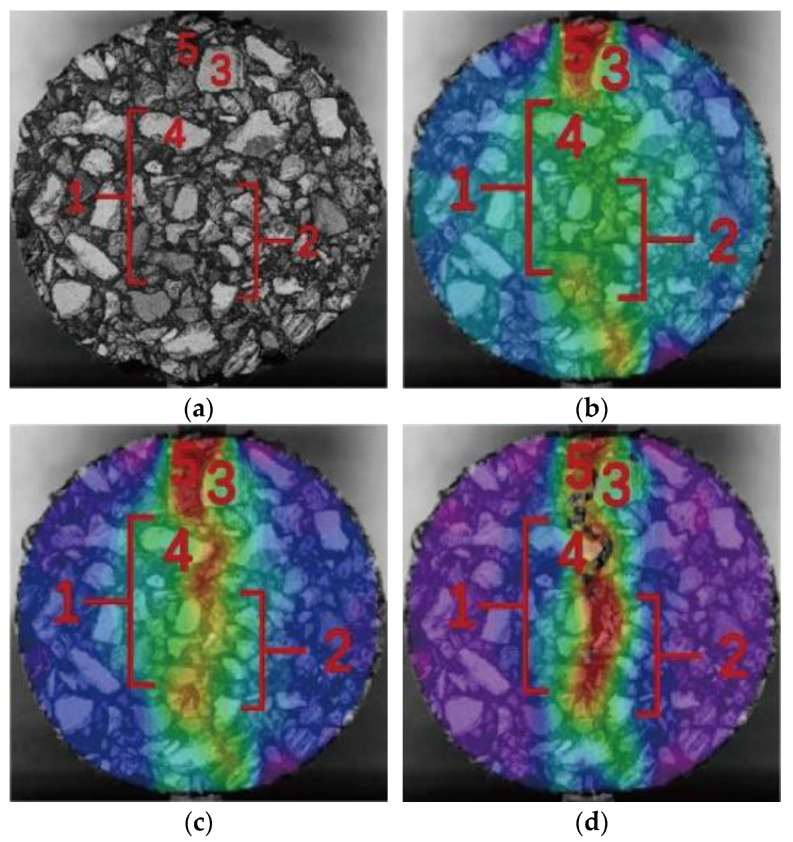
Strain field distribution of specimen: (**a**) original image; (**b**) strain field at point A; (**c**) strain field at point B; and (**d**) strain field at point C.

In order to characterize the fracture behavior of asphalt mixtures, the real-time horizontal tensile strain (*E_xx_*) of different fiber–asphalt mixtures during the cracking process was obtained using digital image correlation technology. Based on the above steps, the strain field distribution images corresponding to the 75% peak load before the peak (point A), the peak load (point B), and the 75% peak load after the peak (point C) in the load–displacement curve were accurately obtained, as shown in Figure 7. Regions 1 and 2 were two strip regions close to the middle of the specimen, regions 3 and 4 were two coarse aggregates in the middle of the specimen, and region 5 was the mortar region at the top of the specimen. These images indicated that the digital image correlation technology had good applicability in the test.

## 3. Results and Discussion

### 3.1. Results of Indirect Tensile Asphalt Cracking Test

#### 3.1.1. Effects of Fiber Content on Cracking Resistance

**Figure 8:**
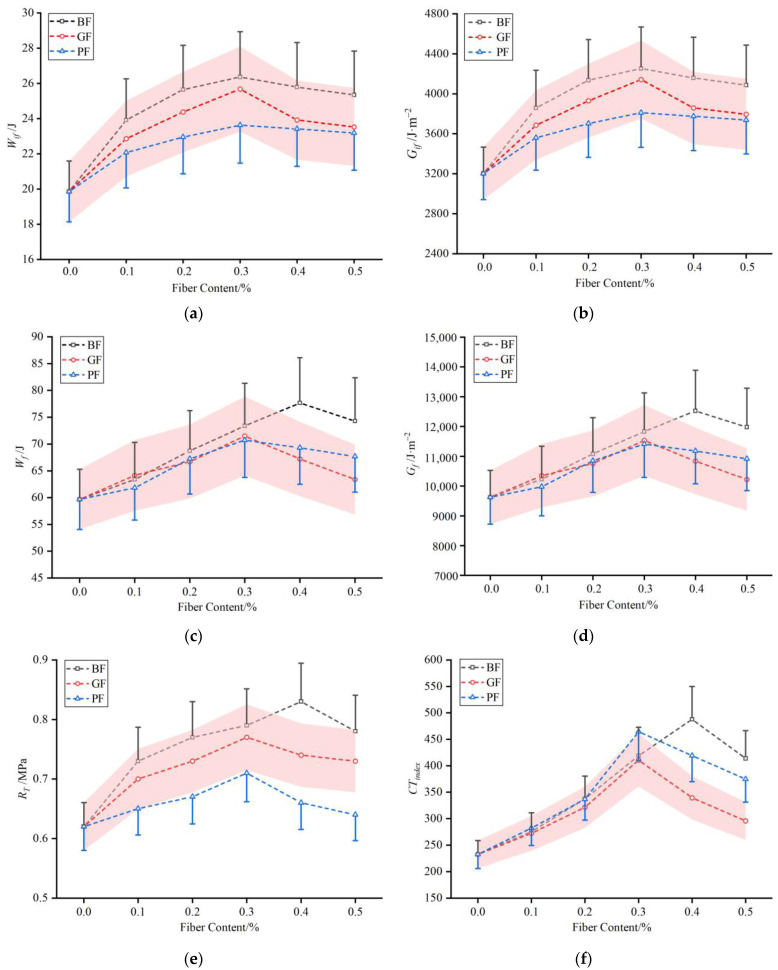
Results of six evaluation indexes: (**a**) *W_if_*; (**b**) *G_if_*; (**c**) *W_f_*; (**d**) *G_f_*; (**e**) *R_T_*; and (**f**) *CT_index_*.

Figure 8 shows the results of six evaluation indexes of fiber–asphalt mixtures from the indirect tensile asphalt cracking test. As seen in Figure 8a,b, the crack initiation work (*W_if_*) and crack initiation energy (*G_if_*) of asphalt mixtures increased to the maximum values when fiber content reached 0.3% for each type of fiber. Specifically, the enhancement of *G_if_* was 32.7%, 29.3%, and 19.0% for BF-, GF-, and PF-modified asphalt mixtures, respectively.

As seen in Figure 8c,d, the crack work (*W_f_*) and crack energy (*G_f_*) of asphalt mixtures increased after adding different content of fibers. For the basalt fiber–asphalt mixture, the enhancement was the largest when the fiber content was 0.4%—the enhancement increased by 30.1% compared with the non-fiber. For glass fiber and polyester fiber–asphalt mixtures, the enhancement was the largest—increased by 19.8% and 18.5%, respectively—when the fiber content was 0.3%.

As seen in Figure 8e, the splitting tensile strength (*R_T_*) of asphalt mixtures increased after adding different content of fibers. For the basalt fiber–asphalt mixture, the enhancement was the largest when the fiber content was 0.4%—the enhancement increased by 33.9% compared with the non-fiber. For glass fiber and polyester fiber–asphalt mixtures, the enhancement was the largest—increased by 24.2% and 14.5%, respectively—when the fiber content was 0.3%.

As seen in Figure 8f, the cracking tolerance index (*CT_index_*) of asphalt mixtures increased after adding different content of fibers. For the basalt fiber–asphalt mixture with a 0.4% fiber content, the enhancement was the largest, increasing by 110.0% compared with the non-fiber. For glass fiber and polyester fiber–asphalt mixtures, the enhancement was the largest—increased by 76.9% and 100.0%, respectively—when the fiber content was 0.3%.

In summary, all indexes of cracking resistance of fiber–asphalt mixtures were increased firstly and then decreased with the increase of fiber content, and there was an optimum content for all three types of fibers. With the increase of the content, the fiber could effectively fulfill its function of bonding, reinforcing, and transferring load in asphalt mixtures, thereby enhancing the cracking resistance [8]. However, after the optimum content was reached, agglomeration and uneven dispersion were more likely to occur due to excessive fiber, which affected the effects of fiber in asphalt mixtures and reduced the performance improvement effect. A. Mrema et al. added glass fiber to a dense-graded asphalt mixture, and the experimental investigation revealed that with the increase of glass fiber content, the indirect tensile strength initially exhibited an increase trend and then decreased [31]. Therefore, it is necessary to consider the dispersion uniformity when mixing various types of fibers into asphalt mixtures. Considering all evaluation indexes comprehensively, it was found that for basalt fiber, glass fiber, and polyester fiber, the cracking resistance of mixtures was highly improved when the fiber content was 0.4%, 0.3% and 0.3%, respectively.

#### 3.1.2. Effects of Fiber Type on Cracking Resistance

**Figure 9:**
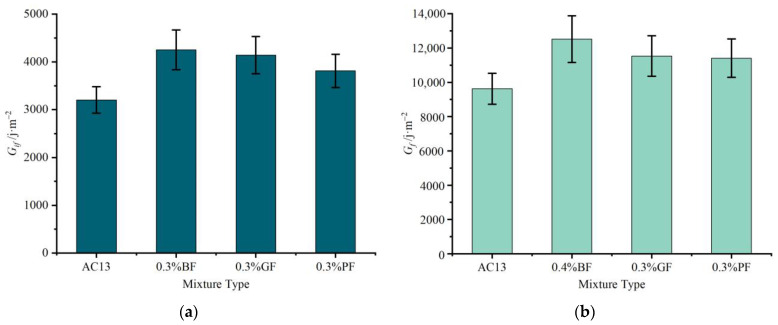

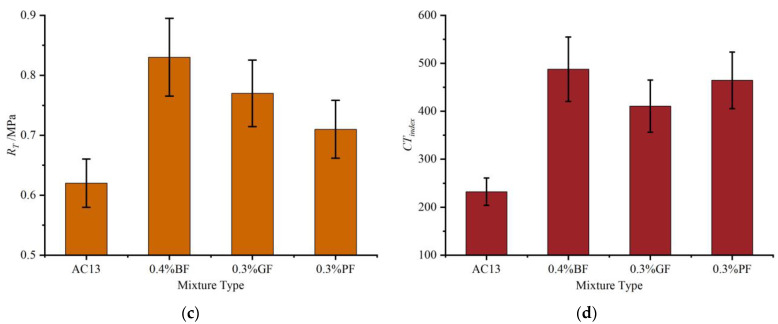
Results of four evaluation indexes at the optimum fiber content: (**a**) *G_if_*; (**b**) *G_f_*; (**c**) *R_T_*; and (**d**) *CT_index_*.

Figure 9 shows the results of four evaluation indexes (*G_if_*, *G_f_*, *R_T_*, and *CT_index_*) at the optimum fiber content. As seen in Figure 9a, at the optimum content, the addition of three types of fibers could increase *G_if_* of asphalt mixtures by more than 19.0%. Basalt fiber presented the best improvement effect, followed by glass fiber and polyester fiber. Fiber possessed a certain degree of binding force on aggregates, preventing them from becoming loose and enhancing the overall integrity of asphalt mixtures. Consequently, fiber played a role in increasing toughness and improving *G_if_*. In addition, the improvement effect of fiber on the cracking initiation resistance of mixtures was affected by the strength of the fiber itself. The break strength of basalt fiber was the highest, followed by glass fiber, and polyester fiber was the lowest. A moderate amount of fiber could be evenly dispersed in mixtures and bonded to each other to form a fiber network. The greater the tensile strength of the fiber, the more effectively it could inhibit crack formation and improve the cracking initiation resistance of mixtures.

As seen in Figure 9b, at the optimum content, the addition of three types of fibers could increase *G_f_* of asphalt mixtures by more than 18.5%. The best enhancement effect was observed in basalt fiber, while glass fiber and polyester fiber exhibited similar enhancement effects. Compared with the other two fibers, basalt fiber presented a remarkable improvement effect on *G_f_* of mixtures.

As seen in Figure 9c, at the optimum content, the addition of three types of fibers could increase *R_T_* of asphalt mixtures by more than 14.5%. The improvement effect on *R_T_* varied greatly among the three types of fibers, and the best improvement effect was achieved with basalt fiber. The type of fiber had a great effect on the deformation resistance of mixtures, and basalt fiber allowed mixtures to withstand greater load when cracking. This was because the improvement effect of fiber on the deformation characteristics of mixtures was affected by the modulus and strength of the fiber.

As seen in Figure 9d, at the optimum content, the addition of three types of fibers could significantly increase *CT_index_* of asphalt mixtures, reaching more than 76.9%. The effects of the three fibers on *CT_index_* was quite different, and basalt fiber presented the best improvement effect. The fiber type had a great effect on the resistance of mixtures to crack expansion, and basalt fiber was more able to improve the resistance. This was because basalt fiber had higher break strength and elastic modulus and a better synergistic effect with asphalt mixtures [32]. Though the *R_T_* value of asphalt mixture with polyester fiber was lower than that with glass fiber, the *CT_index_* value showed the opposite trend. This was due to the better flexibility and larger break elongation of polyester fiber, which could effectively delay the crack expansion of mixtures.

In summary, the fiber type had a great effect on the cracking resistance of mixtures. Basalt fiber could increase *G_if_*, *G_f_*, *R_T_*, and *CT_index_* most effectively, and enhance both the cracking initiation resistance and cracking expansion resistance of asphalt mixtures. This might be attributed to the properties of basalt fiber, which could play a superior toughening role in mixtures, and alleviate the phenomenon of internal stress concentration [33]. The increase in *CT_index_* of the mixture with polyester fiber was greater than that with glass fiber, mainly due to the higher break elongation of polyester fiber, which could better enhance the cracking expansion resistance of mixtures.

### 3.2. Fracture Behavior Characterizations of Mixture

#### 3.2.1. Real-time Tensile Strain Analysis

**Figure 10:**
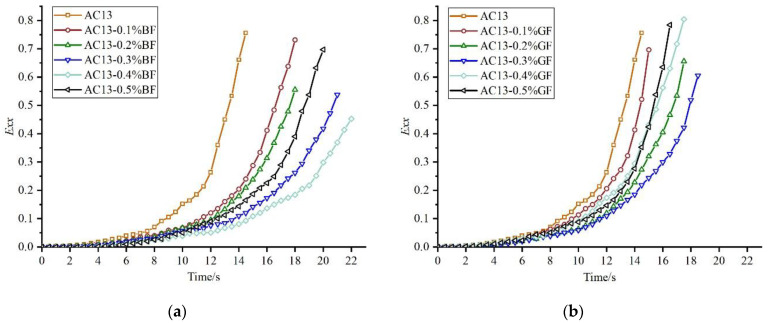

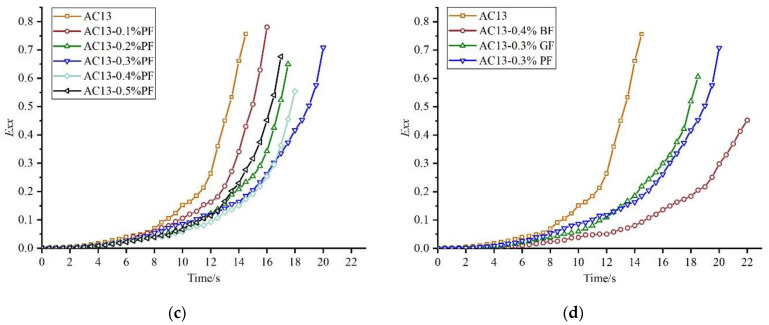
*E_xx_* of fiber–asphalt mixtures: (**a**) AC13-BF; (**b**) AC13-GF; (**c**) AC13-PF; (**d**) Comparison of different types.

In order to study the fracture behavior of fiber–asphalt mixture, digital image correlation technology was used to obtain the real-time horizontal tensile strain (*E_xx_*) of different fiber–asphalt mixtures during the indirect tensile asphalt cracking test, as seen in Figure 10. During the cracking process of mixtures, the real-time horizontal tensile strain exhibited a synchronous increase with the vertical displacement. Prior to reaching the peak load, cracks formed and developed at a slow pace, accompanied by a relatively gradual growth in strain captured using the digital correlation technology. Subsequently, upon reaching the peak load, cracks rapidly expanded until the specimen ultimately failed, accompanied by relatively rapid growth in strain captured using the digital correlation technology. Therefore, the growth of strain could be roughly divided into two stages based on the occurrence of the peak load, as seen in Figure 4. The first one was the slow-growth stage, in which the strain changed approximately linearly with time and grew slowly. The second one was the rapid-expansion stage, in which the strain increased rapidly with time and the crack expanded rapidly until the specimen failed.

As seen in Figure 10a–c, at the same moment, the tensile strain of fiber–asphalt mixtures was smaller than that of non-fiber mixture, and the strain difference increased with time. This indicated that adding fiber could delay the appearance and expansion of cracks and improve the cracking resistance of mixtures. For the basalt fiber–asphalt mixture, the minimum real-time strain and the best cracking delay effect were achieved when the fiber content was 0.4%, followed by the content of 0.3%. For the glass fiber–asphalt mixture, the cracking delay effect was the best when the fiber content was 0.3%, followed by the content of 0.2%. For polyester fiber–asphalt mixture, the cracking delay effect was similar when the fiber content was 0.3% and 0.4%, while the time required for asphalt mixtures to reach failure was longer when the content was 0.3%. In summary, the cracking delay effects of the three types of fibers were poor when the content was 0.1%, and with the increase of the content, the cracking delay effect became more and more obvious; however, the effect was weakened after the optimum content was reached. Overall, fibers did affect the fracture behavior and cracking resistance of mixtures. On the one hand, due to the large specific surface area, fibers can absorb surface asphalt and hold the internal asphalt, resulting in the proportion reduction of free asphalt and proportion increase of structural asphalt [33,34]. Subsequently, the internal bonding force of asphalt mixtures will be effectively improved. On the other hand, the fiber and aggregate are bonded to form a network, which can greatly increase the integrity of the fiber and asphalt mixture in co-deformation, and improve the tensile strength and bearing capacity. Therefore, the appearance and expansion of cracks in the mixture can be delayed.

As seen in Figure 10d, the three fibers could significantly reduce the real-time strain of asphalt mixtures at the optimum content. Among them, the basalt fiber–asphalt mixture showed the best cracking resistance in both stages, while the effect of glass fiber was better than polyester fiber in the first stage, and inferior in the second stage. This was mainly because the strength of polyester fiber was low, and the effect of resistance to crack appearance was weaker than the other two fibers. However, due to the larger break elongation, polyester fiber could play a greater role in the resistance to crack propagation in the rapid-expansion stage. R. Hong et al. found that polyester fiber with high break elongation could effectively enhance the toughness and delay the crack expansion of asphalt mixtures [35]. It can be seen that due to the differences in physical properties of fibers themselves, the fracture behavior of different fiber–asphalt mixtures was also affected to a certain extent. Basalt fiber had higher break strength and modulus, which could better delay the appearance and expansion of cracks and improve the cracking resistance of mixtures.

#### 3.2.2. Determination of Fracture Parameters

In order to quantitatively characterize the fracture behavior of fiber–asphalt mixtures, the real-time tensile strain curves of mixtures were fitted by stages. The real-time tensile strain curve of each fiber–asphalt mixture was approximately linear in the slow-growth stage, while in the rapid-expansion stage, the strain changed exponentially with time. The formulas of the fitting model are shown in Equations (4) and (5), and the fracture parameters of *k*_1_ and *k*_2_ were obtained.

(4) $$E_{xx}^{1}=k_{1}t+b$$

(5) $$E_{xx}^{2}=a\times \exp\left(k_{2}t\right)$$

where $E_{xx}^{1}$ is the real-time strain of the slow-growth stage; $E_{xx}^{2}$ is the real-time strain of the rapid-expansion stage; *t* is the cracking time; *k*_1_ and *k*_2_ are fracture parameters; and *a* and *b* are fitting parameters.

**Figure 11:**
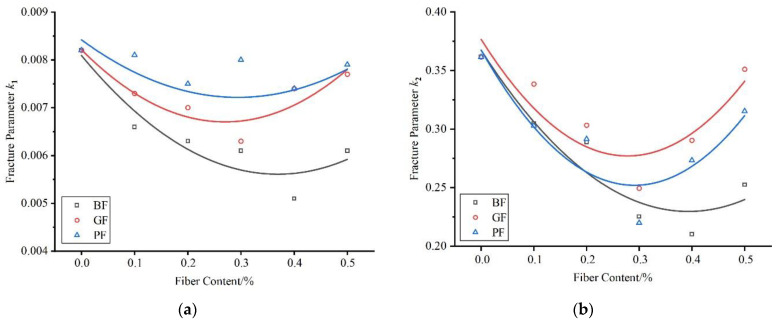
Fracture parameters of fiber–asphalt mixtures: (**a**) fracture parameter *k*_1_; (**b**) fracture parameter *k*_2_.

In the formula, *k*_1_ and *k*_2_ can reflect the strain growth rate of the two stages, respectively. The smaller the values, the better the cracking resistance of the mixture achieved. The fracture parameters of each fiber–asphalt mixture are shown in Figure 11. As seen in Figure 11, the addition of fiber could reduce the fracture parameter values and improve the cracking resistance of mixtures to different degrees. The asphalt mixture with 0.4% basalt fiber content had the smallest *k*_1_ and *k*_2_ values, indicating the best ability to delay crack appearance and expansion. The fracture parameter *k*_1_ of polyester fiber in the slow-growth stage was higher than that of glass fiber, while the fracture parameter *k*_2_ in the rapid-expansion stage was lower than that of glass fiber, which indicated that polyester fiber had a better cracking delay effect during crack rapid expansion.

#### 3.2.3. Cracking Time Analysis

**Figure 12:**
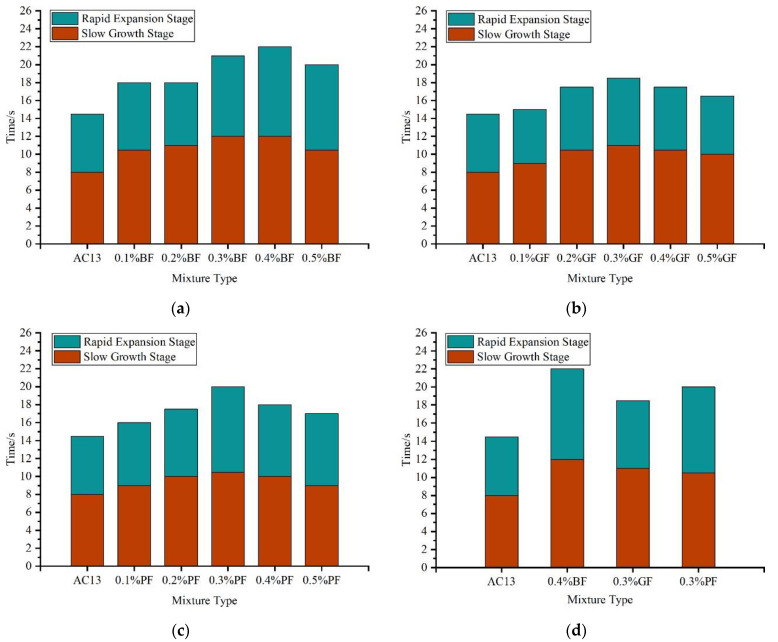
Time of each stage: (**a**) AC13-BF; (**b**) AC13-GF; (**c**) AC13-PF; (**d**) Comparison of different types.

Slow-growth-stage time, rapid-expansion-stage time, and total cracking time were obtained to further study the fracture behavior of fiber–asphalt mixtures. As mentioned above, the corresponding time of peak load was taken as the dividing point of the two stages, and the results are shown in Figure 12.

The longer the duration of the slow-growth stage, the better the fiber at delaying crack appearance, and the tensile strain of asphalt mixtures at this stage was approximately linear elasticity. The longer the duration of the rapid-expansion stage, the better the fiber at delaying crack expansion, and the plastic fracture occurred in mixtures at this stage. The total time of the two stages was the failure time of mixtures, which could reflect the whole cracking resistance of mixtures to a certain extent.

As seen in Figure 12a–c, the addition of each fiber extended the cracking time at both stages and the failure time of asphalt mixtures to varying degrees, which indicated that fiber could play a role in resisting the occurrence of cracks and delaying the expansion of cracks in mixtures, and improving the cracking resistance of mixtures. For the basalt fiber–asphalt mixture, when the fiber content was 0.4%, the time duration in both the slow-growth stage and rapid-expansion stage of cracks was the longest, indicating the best cracking delay effect. For glass fiber and polyester fiber–asphalt mixtures, the time duration in both stages was the longest when the content was 0.3%. With the increase of fiber content, the effect of delaying crack became more and more obvious, until an optimum content was achieved.

As seen in Figure 12d, all three types of fibers could significantly prolong the failure time of asphalt mixtures at the optimum content. The cracking delay effect of the basalt fiber–asphalt mixture was the best in both stages, while the delay effect of glass fiber, in comparison with that of polyester fiber, was better in the slow-growth stage, but worse in the rapid-expansion stage. This is consistent with the results of real-time tensile strain of fiber–asphalt mixtures. In terms of failure time, three types of fibers were shown in the following order: basalt fiber, polyester fiber, and glass fiber.

#### 3.2.4. Cracking Path Analysis

**Figure 13:**
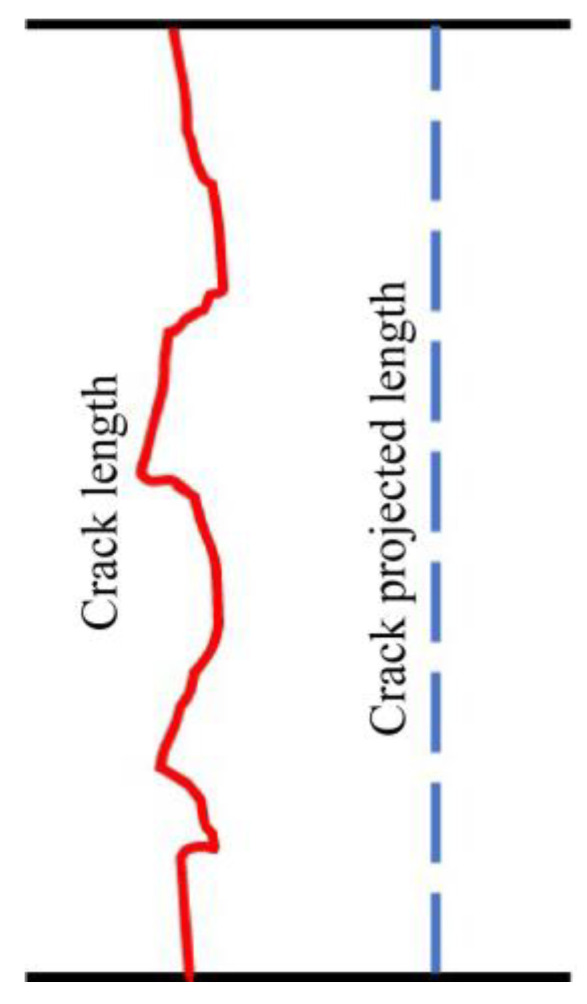
Diagram of the crack description.

In order to study the influence of fiber on the cracking path of asphalt mixtures, some researchers have put forward the index of crack tortuosity (*C_T_*) [36,37]—the larger the value, the better the cracking resistance. The calculation formula is shown in Equation (6).

(6) $$C_{T}=L_{m}/L_{P}$$

where *C_T_* is the crack tortuosity; *L_m_* is the crack length, mm; and *L_p_* is the crack projected length, mm, as seen in Figure 13.

**Figure 14:**
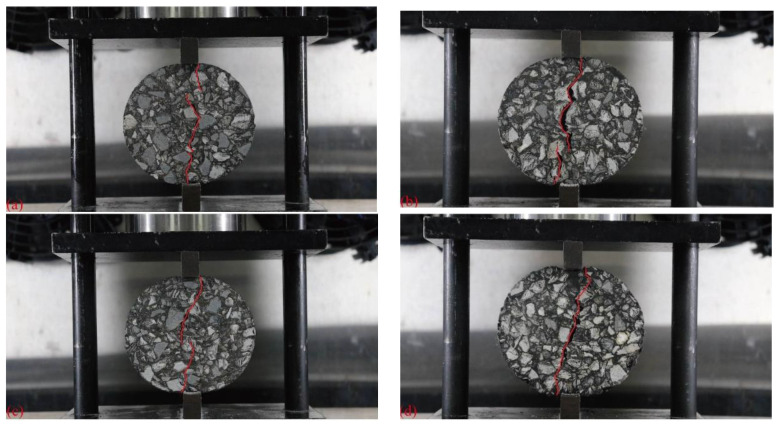
Cracking path of fiber–asphalt mixtures: (**a**) AC13; (**b**) AC13-0.4%BF; (**c**) AC13-0.3%GF; and (**d**) AC13-0.3%PF.

The effects of basalt fiber, glass fiber, and polyester fiber on the cracking path of mixtures was studied at the optimum content of 0.4%, 0.3%, and 0.3%, respectively. As shown in Figure 14, the corresponding images at the failure point of the specimens were synchronously collected during the indirect tensile asphalt cracking test, and the crack length was identified using image technology. The results of *C_T_* are shown in Table 4.

As can be seen from Figure 14 and Table 4, after adding different fibers, the cracking paths of mixtures were extended and the *C_T_* values increased. This might be because the incorporation of fibers could enhance the toughness and strength of asphalt binder, thereby guiding cracks to propagate along the surface of coarse aggregates. It reflected that adding fiber delayed the expansion of cracks, thereby enhancing the cracking resistance of mixtures. Compared with the non-fiber, the *C_T_* values of asphalt mixtures with basalt fiber, glass fiber, and polyester fiber increased by 9.5%, 6.0%, and 4.3% respectively.

## 4. Conclusions

In this research, different types (basalt fiber, glass fiber, and polyester fiber) and content (0.1%, 0.2%, 0.3%, 0.4%, and 0.5%) were selected to prepare AC13 asphalt mixtures. Based on an indirect tensile asphalt cracking test, the effects of fiber on the cracking resistance of mixtures were studied. Combined with digital image correlation technology, the influence of fiber type and content on the fracture behavior of mixtures was further studied, and the conclusions are as follows:

1. Adding fiber can improve the cracking resistance of asphalt mixtures, and different fiber demonstrates the superior enhancing effect at a certain optimum content, which is 0.4%, 0.3%, and 0.3% for BF, GF, and PF, respectively.
2. In terms of fiber type, basalt fiber presents the best-enhancing effect on the cracking resistance of mixtures at the optimum content.
3. As a type of organic fiber, PF shows an excellent function in improving the cracking expansion resistance, due to its high break elongation.
  - The real-time tensile strain curve during the cracking process can be obtained using DIC technology, based on which the fracture parameters *k*_1_ and *k*_2_ can be proposed and be used to quantitatively evaluate the fracture behavior of fiber–asphalt mixtures.
4. Based on DIC technology, adding each type of fiber can effectively inhibit strain development, prolong the cracking duration time, and extend the cracking path. In addition, BF also presents a superior enhancing effect.

This research only focused on the indirect tensile asphalt cracking test to evaluate the performance of fiber–asphalt mixtures; more testing methods can be used in the future. In addition, only three types of fibers were selected, and future investigations could explore the impacts of more types of fibers on the fracture behavior of mixtures.

## Figures and Tables

**Table 1:** Indexes of SBS-modified asphalt.

Index		Result
Softening point/°C		77
Penetration (25 °C)/0.1 mm		51.8
Ductility (5 cm/min, 5 °C)/cm		30
Recovery of elasticity (25 °C)/%		79
RTFOT residue	Weight change/%	−0.07
	Penetration ratio/%	88
	Residual ductility (15 °C)/cm	32

**Table 2:** Indexes of different types of fibers.

Index	BF	GF	PF
Break strength/MPa	2835	2726	921
Break elongation/%	5.70	4.83	36.48
Elastic modulus/GPa	90	75	32
Apparent density/g·cm^−3^	2.49	2.50	1.32
Melting point/°C	1500	1500	253

**Table 3:** Optimum asphalt content of fiber–asphalt mixtures.

Gradation Type	Fiber Content/%	Optimum Asphalt Content/%		
		BF	GF	PF
AC13	0	4.9	4.9	4.9
	0.1	4.9	4.9	5.0
	0.2	5.0	5.0	5.1
	0.3	5.1	5.1	5.2
	0.4	5.2	5.1	5.2
	0.5	5.2	5.2	5.3

**Table 4:** *C_T_* of fiber–asphalt mixtures.

Index	AC13	AC13-0.4%BF	AC13-0.3%GF	AC13-0.3%PF
*L_m_*/mm	90.113	96.732	93.579	93.409
*L_P_*/mm	87.090	85.340	85.268	86.489
*C_T_*	1.035	1.133	1.097	1.080

## Data Availability

Not applicable.

## Abbreviations

**Abbreviations**	**Non-Abbreviated Forms**
DIC	Digital image correlation
IDEAL-CT	Indirect tensile asphalt cracking test
BF	Basalt fiber
GF	Glass fiber
PF	Polyester fiber
*W_if_*	Crack initiation work
*W_f_*	Crack work
*G_if_*	Crack initiation energy
*G_f_*	Crack energy
*R_T_*	Splitting tensile strength
*CT_index_*	Cracking tolerance index
*E_xx_*	Real-time tensile strain
*C_T_*	Crack tortuosity
*L_m_*	Crack length
*L_p_*	Crack projected length
